# Decreasing the Crystallinity and Degree of Polymerization of Cellulose Increases Its Susceptibility to Enzymatic Hydrolysis and Fermentation by Colon Microbiota

**DOI:** 10.3390/foods12051100

**Published:** 2023-03-04

**Authors:** Karel Thielemans, Yamina De Bondt, Luke Comer, Jeroen Raes, Nadia Everaert, Bert F. Sels, Christophe M. Courtin

**Affiliations:** 1 Laboratory of Food Chemistry and Biochemistry, Department of Microbial and Molecular Systems (M^2^S), KU Leuven, Kasteelpark Arenberg 20, 3001 Leuven, Belgium; 2 Center for Sustainable Catalysis and Engineering, Department of Microbial and Molecular Systems (M^2^S), KU Leuven, Celestijnenlaan 200F, 3001 Leuven, Belgium; 3 Divison Animal and Human Health Engineering, Department of Biosystems, KU Leuven, Kasteelpark Arenberg 30, 3001 Leuven, Belgium; 4 Department of Microbiology and Immunology, KU Leuven, Herestraat 49–Bus 1028, 3000 Leuven, Belgium

**Keywords:** cellulose, depolymerization, amorphization, dietary fiber, prebiotic, colonic fermentation, short chain fatty acids

## Abstract

Cellulose can be isolated from various raw materials and agricultural side streams and might help to reduce the dietary fiber gap in our diets. However, the physiological benefits of cellulose upon ingestion are limited beyond providing fecal bulk. It is barely fermented by the microbiota in the human colon due to its crystalline character and high degree of polymerization. These properties make cellulose inaccessible to microbial cellulolytic enzymes in the colon. In this study, amorphized and depolymerized cellulose samples with an average degree of polymerization of less than 100 anhydroglucose units and a crystallinity index below 30% were made from microcrystalline cellulose using mechanical treatment and acid hydrolysis. This amorphized and depolymerized cellulose showed enhanced digestibility by a cellulase enzyme blend. Furthermore, the samples were fermented more extensively in batch fermentations using pooled human fecal microbiota, with minimal fermentation degrees up to 45% and a more than eight-fold increase in short-chain fatty acid production. While this enhanced fermentation turned out to be highly dependent on the microbial composition of the fecal pool, the potential of engineering cellulose properties to increased physiological benefit was demonstrated.

## 1. Introduction

Cellulose is the most abundant renewable material in nature, being the primary building block of the plant cell wall. It consists of long unbranched β-1,4-bound glucose polymers organized in long crystalline fibers with strong interactions between the different polymers [1]. The high degree of crystallinity, high degree of polymerization and low specific surface give cellulose a very recalcitrant character. This recalcitrance is important in the plant cell wall, since cellulose provides the plant with mechanical strength and resilience against breakdown, but is a major drawback in valorization strategies of (ligno)cellulose, e.g., in the context of biorefineries [1,2]. Furthermore, cellulose acts as an insoluble and recalcitrant dietary fiber in the human body when ingested.

The use of cellulose as dietary fiber in foods could be very relevant since the food industry increasingly searches for dietary fiber enrichment strategies. While a sufficient daily intake of dietary fiber is correlated with various health benefits, such as a decreased risk of colorectal cancer, obesity, cardiovascular diseases and diabetes mellitus type II [3,4,5,6], the average daily intake of dietary fiber is too low in Western diets [7]. Within dietary fiber fortification strategies, specific attention goes to (partially) fermentable dietary fiber. Fermentation of dietary fiber in the colon is correlated with different additional physiological benefits, linked to the production of short-chain fatty acids (SCFA), which are essential for colonic health h, glucose and cholesterol homeostasis and the regulation of the appetite [8,9,10]. The fermentability of cellulose in the human colon is very low, however [11]. Cellulolytic enzymes are produced by *Ruminococcus*, *Enterococcus*, *Bacteroides* or *Prevotella* species in the colon [12,13], but the highly ordered nature of cellulose limits the accessibility of the cellulosic fibers and the glucosidic β-1,4 bonds for enzymatic breakdown and results in very limited fermentability.

We can assume that the fermentability and the physiological benefits of cellulose with it could be improved by breaking this recalcitrance before ingestion. Such accessible cellulose would remain insoluble and indigestible but could be fermented to a greater extent in the colon. Previous research already stated that the fermentability of cellulose depends on its physical appearance [14], and some attempts to improve cellulose fermentability by reducing the particle size were already successful in human in vitro experiments [15,16]. However, the impact of the degree of polymerization and crystallinity has not been investigated in this context. At the same time, these structural parameters are known to affect cellulose accessibility greatly [17,18,19].

Plenty of (ligno)cellulosic biomass pretreatment protocols, such as milling, irradiation, ultrasonication, hydrothermal treatment or solubilization in ionic liquids, have been developed and optimized to alter these cellulose characteristics [17,20,21,22,23,24,25]. Moreover, several of them, such as ball milling, acid hydrolysis or solubilization in ionic liquid, are linked to an improved cellulose enzymatic accessibility as well [26,27,28]. In this study, two effective pretreatment methods, ball milling and acid hydrolysis, are combined to make cellulose with a lowered degree of polymerization, a lowered degree of crystallinity and the combination of both. These samples are used to gain insight into the effect of these parameters on the enzyme accessibility of cellulose and its fermentability by colon microbiota, using batch in vitro fermentations.

## 2. Materials and Methods

### 2.1. Materials

Microcrystalline cellulose (Avicel PH-101, 3.4% moisture), citric acid (analytical grade), the Cellic CTec2 cellulase enzyme blend and all other analytical chemicals and solvents were purchased from Sigma-Aldrich (Deurne, Belgium).

### 2.2. Production of Dietary Fiber Samples Starting from Microcrystalline Cellulose

**Figure 1:**
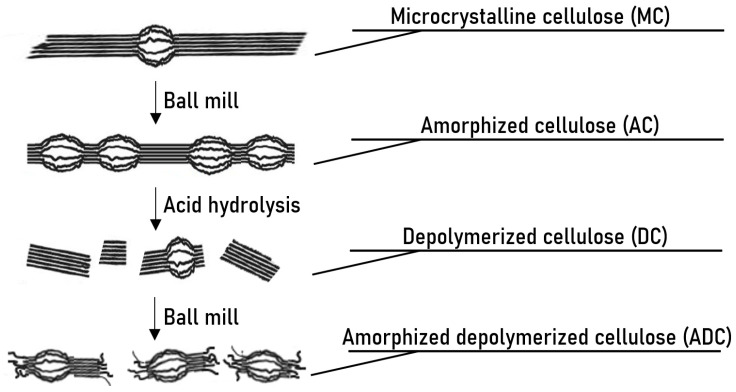
Visual representation of the structural changes during the production process of the different fibers, starting from microcrystalline cellulose (MC), through amorphized cellulose (AC) and depolymerized cellulose (DC), to give amorphized depolymerized cellulose (ADC). Closely packed polymers represent crystalline zones, while paracrystalline zones are visualized by loosely bound polymers.

An overview of the production of the different dietary fiber samples is given in Figure 1 and Table A1. Microcrystalline cellulose (MC) was first depolymerized using a ball milling step and acid hydrolysis, similar to our previous work [29]. MC was pretreated in a planetary ball mill (PM100, Retsch GmbH, Haan, Germany) in batches of 20 g with 6 zirconium oxide balls (Ø 10 mm) to induce para-crystalline zones in the cellulose fiber. These ball-milled cellulose fibers are called amorphized cellulose (AC). Milling time (60–360 min) and speed (400–500 rpm) were varied. Afterwards, the paracrystalline zones in the AC fibers were hydrolyzed with a 10% citric acid solution in water. Hydrolysis time (2–6 h) and temperature (90–130 °C) were varied (Table A1). The depolymerized insoluble cellulose samples were washed until neutral pH and dried for 45 h at 60 °C, yielding depolymerized cellulose (DC). After being dried, the DC sample was again treated in the planetary ball mill with 6 zirconium oxide balls (Ø 10 mm) at 500 rpm to produce amorphized depolymerized cellulose (ADC). Treatment times of 30, 60 and 360 min were used. All samples and respective production parameters are summarized in Table A1.

### 2.3. Characterization of Dietary Fiber Samples

The average degree of polymerization (avDP) of cellulose was determined viscometrically in triplicate, based on the method of the French Institute for Normalisation [30]. Fiber samples (0.075 g) were dissolved in a 0.5 M bis(ethylenediamine)copper(II)hydroxide solution (15 mL), and the viscosity of this solution at 25 °C was measured with a capillary viscometer, type nr. 509 04 (Schott Geräte, Jena, Germany). The avDP was calculated from the boundary viscosity of the solution (η), based on the empirical relation: Average DP^α = η/K, with α and K empirical constants, equal to 1 and 7.5 × 10^−3^, respectively. The boundary viscosity η was determined from η_a_ = η.C.10^((0,14.η.C)), with η_a_ the specific viscosity of the solution, and C the cellulose concentration (g/mL).

The crystallinity of the fiber samples was determined by X-ray powder diffraction (XRD) on a high-throughput STOE STADI P Combi diffractometer (STOE & Cie GmbH, Darmstadt, Germany) in transition mode with Ge(111) monochromatic X-ray inlet beams (λ = 1.5406 Å, Cu Kα source). Crystallinity indexes were determined by the peak-height method of Segal and coworkers [31].

### 2.4. Enzymatic Digestibility Analysis

The enzymatic digestibility of the dietary fiber samples was determined by calculating the enzymatic conversion (EC) after incubating samples with the Cellic CTec2 cellulase enzyme blend, as described by Chen and coworkers [32]. Cellulose was suspended (1.0% *w*/*v*) in a 50 mM sodium acetate buffer (pH 4.8) with 20 U Cellic CTec2 cellulase enzyme blend per gram cellulose and stirred at 900 rpm. After 1 h of incubation at 40 °C, the enzymes were denatured by heating the solution (5 min, 110 °C). The solid fraction was separated from the supernatant by centrifuging at 5000 g. The amount of glucose and cellobiose in the supernatant from cellulose hydrolysis was determined by high-performance-anion-exchange chromatography with pulsed amperometric detection (HPAEC-PAD) on a Dionex ICS3000 chromatography system (Sunnyvale, CA, USA). Saccharides were separated on a Dionex CarboPac PA-100 column (4 × 250 mm), equilibrated with 90 mM NaOH. The enzymatic conversion was calculated from the amount of glucose (m_g_) and cellobiose (m_cb_) in the supernatant, and the amount of starting substrate (m_c_):

(1) $$EC=\frac{m_{g}+m_{cb}}{1.1m_{c}}$$

### 2.5. In Vitro Fermentation of Dietary Fiber Samples Using Human Fecal Inoculum

In vitro fermentation experiments (trial 1, 2 and 3) were performed as described by De Preter et al. [33]. Fresh fecal samples of 8 healthy donors (consuming a mixed western diet, no history of antibiotic use in the last six months) were collected and pooled to make a 10 *w*/*v*% fecal slurry in phosphate-buffered saline. After intensive shaking, this fecal slurry was decanted, and the supernatant (referred to as the inoculum) was added to different fiber samples (25 mL to 100 mg cellulose) in triplicate. After being flushed with nitrogen gas, the tubes were incubated anaerobically for 48 h in a shaking water bath at 37 °C. At the end of incubation, the pH of the slurry was measured with a digital pH meter (Hanna Instruments HI 9025, Temse, Belgium). Aliquots were stored at −20 °C for the determination of short-chain fatty acid (SCFA) concentration and microbial analysis.

### 2.6. Short-Chain Fatty Acid Analysis

The amounts of acetate, propionate and butyrate in the fecal inoculum were determined according to the gas-chromatographic method described by Bautil et al. [34]. In this procedure, a 25% (*w*/*v*) NaOH solution was added to the inoculum to create sodium salts of the SCFA, which were neutralized by adding a 50% sulfuric acid solution afterwards. These salts were extracted to a diethyl ether phase, which was analyzed with an Agilent 6890 Series gas chromatograph with an EC-1000 Econo-Cap column (25 m × 0.53 mm, 130 °C, 1.2 μm film thickness) and helium (20 mL/min) as carrier gas. A flame ionization detector at 195 °C measured the different fatty acids. Within this analysis, 2-ethyl butyric acid was used as an internal standard.

### 2.7. Microbial Analysis

Microbial profiling was done as described by Falony et al. [35]. Nucleic acids were extracted from the aliquots using the RNeasy PowerMicrobiome kit (Qiagen, Venlo, The Netherlands). The manufacturer’s protocol was modified by adding a heating step at 90°C for 10 min and excluding DNA removal steps. Afterwards, the extracted DNA was amplified in triplicate using 16S primers 515F (59-GTGYCAGCMGCCGCGGTAA-39) and 806R (59-GGACTACNVGGGTWTCTAAT-39) targeting the V4 region. Deep sequencing was performed on a MiSeq platform (2-by-250 paired-end [PE] reads; Illumina, San Diego, CA, USA). Initial quality assessment, sequence filtering and trimming of the FASTQ files were carried out using the FASTQC software (version 0.11.9) and the ‘filterAndTrim’ function of the DADA2 algorithm pipeline package. Analysis thereafter was performed using the ‘mergePairs’ function of the DADA2 package, which merges the forward and reverse sequences. Any chimeric sequences which were produced during aberrant PCR annealing were identified and removed. Taxonomy was assigned to the sequences using a naïve Bayesian classifier method with the SILVA database (version 138.1) as a reference.

### 2.8. Statistics

Significant differences were detected by performing a one-way analysis of variance (ANOVA) using JMP Pro 16 (SAS institute), with a comparison of the mean values using the Tukey test (α < 0.05).

## 3. Results and Discussion

### 3.1. Production of Samples with Different DP and Crystallinity from Microcrystalline Cellulose

To investigate the impact of crystallinity and avDP on the enzymatic accessibility and fermentability by colon microbiota, a modification protocol using the combination of planetary ball mill treatments and acid hydrolysis was used (Figure 1). First, MC was treated in a ball mill to decrease the crystallinity of the cellulose by incorporation of paracrystalline zones. This decrease in crystallinity impacts the levelling-off degree of polymerization (LODP) of the cellulose, which represents the length of crystalline polymers that remain insoluble after a fast hydrolysis of the easily accessible paracrystalline zones [36]. Second, the ball-milled, or amorphized cellulose (AC) was hydrolyzed with citric acid at elevated temperature (90–130 °C) to hydrolyse the polymers in the paracrystalline zones. After this hydrolysis, depolymerized cellulose (DC) with a decreased avDP is obtained. At last, this DC was treated in the planetary ball mill another time for 30–360 min to produce amorphized depolymerized cellulose (ADC), which is expected to be highly accessible. All samples are listed in Table A1.

The influence of processing conditions (ball mill speed/time and acid hydrolysis time/temperature) on cellulose properties was extensively investigated in our previous study for the production of DC [29]. The avDP of the DC fibers can be finely tuned, and also the crystallinity of the DC fibers can be controlled by applying varying process parameters. In short, when the acid hydrolysis is not performed for long enough to hydrolyse all the paracrystalline zones of the AC fibers, the LODP will not be reached and the crystallinity of the resulting DC fibers will remain low [29]. Despite the extensive investigation of the impact of process parameters to produce DC, the impact of the second ball mill treatment on this DC was not yet investigated.

**Figure 2:**
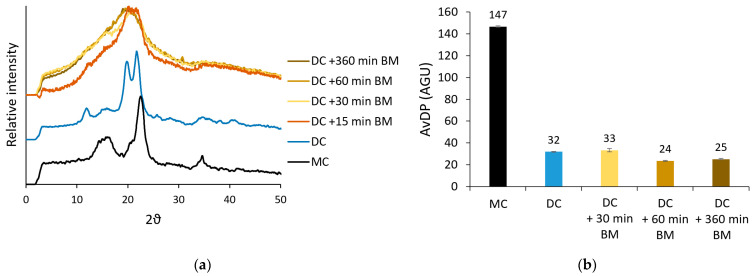
Effect of the ball mill (BM) treatment time (500 rpm) on the (**a**) crystallinity and (**b**) average degree of polymerization (avDP) of depolymerized cellulose (DC). Error bars represent the standard deviation of the analysis. The starting material, microcrystalline cellulose (MC), is included in both figures as a reference.

For a sample with relatively high crystallinity (DC with avDP of 32 AGU and crystallinity index of 0.62), the effect of this additional ball mill treatment on avDP and crystallinity is shown in Figure 2. Figure 2a shows that the peaks from crystalline planes in the refractogram of the DC fibers indeed disappeared due to the ball mill treatment. Milling a DC for only 15 min already disrupted most of the crystalline structure, but the crystalline reflection at 2θ of 22° was still more prominent in these refractograms than in those of ADC fibers with longer milling times. After 30 min of milling of the DC at 500 rpm, an amorphous refractogram was detected, of which the shape did not change anymore upon longer milling times.

**Figure A1:**
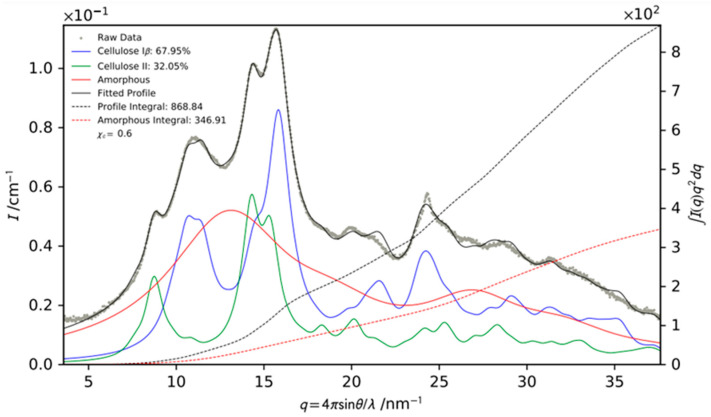
Rietveld refinement analysis of an XRD refractogram of depolymerized cellulose (DC) with an avDP of 32AGU.

Previously, it was shown that the crystallinity decrease during ball milling of unmodified MC was limited during the first 30 min of the milling process [29]. The breakdown of crystallites, therefore, occurred more slowly for unmodified MC than for this DC (Figure 2a). This faster decrease in crystallinity for the DC might be due to the different type of crystallites that need to be broken down. As visualized in Figure A1, 32% of the crystallites in DC fibers were cellulose II polymorphs, while it is known that no cellulose II is present in unmodified MC [37]. We can hypothesize that cellulose II crystallites, formed during the first ball mill treatment and hydrolysis [38], are easier to decrystallize than cellulose I crystallites. The faster decrystallization of DC can also be caused by the lower avDP of the DC fibers.

Depolymerization of the DC fibers does not seem to occur during the ball mill treatment since no significant decrease in avDP was detected for the different ADC fibers (Figure 2b). Previous research stated that a ball mill treatment could not depolymerise cellulose shorter than 50 AGU [29]. This theory seems to be confirmed here since no depolymerization of the DC fibers (DP 32) occurred.

### 3.2. Influence of Cellulose Structural Properties on Enzymatic Accessibility

**Figure 3:**
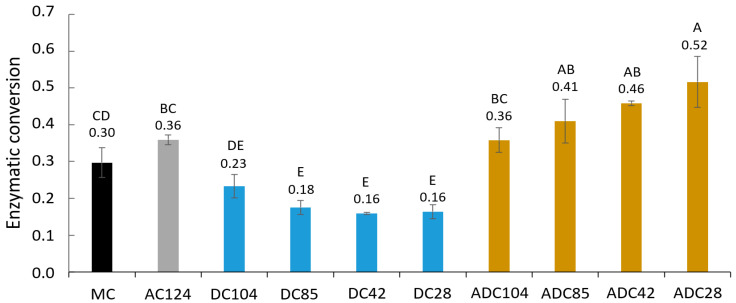
Enzymatic conversion degree after 1 h hydrolysis with Cellic CTec2 enzyme blend for microcrystalline cellulose (MC), amorphized cellulose (AC124), depolymerized cellulose (DC) and amorphized depolymerized cellulose (ADC) with a different average degree of polymerization. ACxx, DCxx or ADCxx represents AC, DC or ADC with an average degree of polymerization of xx. Different letters indicate significant differences (*p* < 0.05).

Figure 3 shows the enzymatic conversion of the modified cellulose into glucose or cellobiose after 1 h reaction with the commercial Cellic CTec2 enzyme blend under optimal conditions. Unmodified MC is compared with amorphized MC (AC124), DC and ADC with different avDP (Table A1). Only 30% of the long crystalline MC was converted into glucose and cellobiose by the cellulase blend within one hour (conversion degree of 0.30 ± 0.04). Decreasing the crystallinity by ball milling (260 min) improved the conversion degree slightly to 0.35 ± 0.01, but decreasing the avDP had the opposite effect. Surprisingly, the DC was all less accessible for the enzyme blend than unmodified MC or AC, while Kumar and Wyman showed that a shorter DP results in higher accessibility [39]. We can hypothesize that a decrease in avDP from 168 to 28 AGU is not sufficient to compensate for the removal of para-crystalline zones and the presence of cellulose polymorph II in the DC fibers, two structural properties that lower enzymatic accessibility. This hypothesis can be confirmed by the positive association between avDP and enzymatic digestibility of the different DC samples. These various DC samples also slightly differed in crystallinity: the crystallinity of DC104 was lower than the crystallinity of DC28, since the LODP was not reached for the longer DC fibers (Table A1). This is because the mildest hydrolysis conditions were used for making DC104, resulting in the remaining of some easily accessible paracrystalline zones after drying. It seems that these small differences in crystallinity have a more significant impact on enzymatic conversion than the differences in avDP.

Since the DC samples showed lower enzymatic digestibility for the cellulase blend than MC or AC, it can be concluded that a DP decrease to values lower than 100 AGU is not of interest to increase the enzymatic accessibility of cellulose. However, this DP decrease pays off once the short cellulose is made amorphous again in the ball mill. ADC with an avDP of 28 AGU had a conversion degree after 1 h of 0.52 ± 0.07, higher than the AC sample. Furthermore, there seems to be a negative correlation between the avDP and enzymatic digestibility for these amorphous samples. Even within the small DP range of 20 to 110 AGU, shortening the cellulose avDP enhances its enzymatic digestibility once a low crystallinity is assured.

### 3.3. Effect of Enhanced Accessibility of Cellulose on Fermentation in the Human Colon

**Figure 4:**
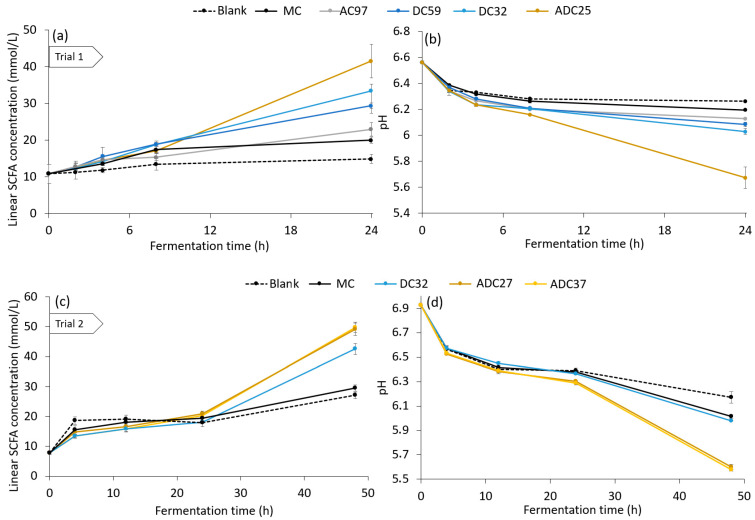

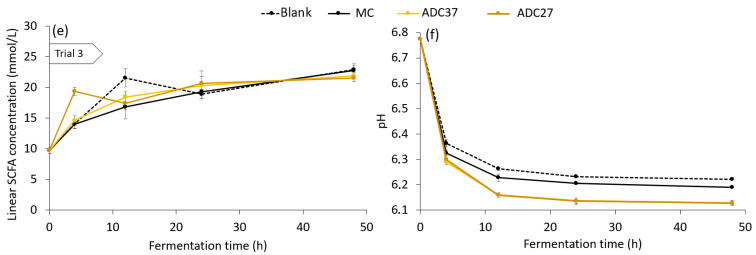
Linear short-chain fatty acid (SCFA) concentration (**a**,**c**,**e**) and pH (**b**,**d**,**f**) of cellulose-enriched fecal inocula in three independent batch in vitro fermentations with pooled human feces, in function of incubation time. ACxx, DCxx or ADCxx represents the fecal inoculum with an amorphized cellulose, depolymerized cellulose or amorphized depolymerized cellulose with an average DP of xx added.

A correlation between the enzymatic accessibility of cellulose samples for the Cellic CTec2 enzyme blend and the fermentability by colon microbiota can be expected since the fermentation of complex carbohydrates starts with hydrolysis by excreted microbial hydrolytic enzymes as well [40]. The behaviour of the fiber samples in the human large intestine was evaluated in three independent batch fermentation experiments using fecal inocula. In Figure 4, the production of linear SCFA and the pH evolution during each fermentation experiment are shown. In these experiments, MC, AC, DC and ADC with different avDP were added to the fecal inocula (Table A1).

In trial one (Figure 4a,b), only a limited amount of linear SCFA was produced in the fecal inoculum without cellulose addition (blank) during the incubation time of 24 h. Adding dietary fiber samples to the inoculum, however, resulted in enhanced production of SCFA during incubation. The majority of SCFA was only produced after the first 8 h of incubation had passed. As described by Mikkelsen et al., cellulose fermentation in a batch in vitro system is slow compared to other readily fermentable carbohydrates, such as arabinoxylans and glucans [14]. In this secondary fermentation phase, it became clear that only a limited amount of MC was fermented within 24 h. During the incubation of MC, the linear SCFA concentration only increased from 10.83 ± 2.63 mmol/L to 19.99 ± 0.71 mmol/L. Breaking cellulose crystallinity by ball milling increased the fermentability already slightly. The average SCFA production after 24 h from the AC sample was 0.57 times higher than the SCFA production from MC. Decreasing the avDP of cellulose was a more effective way to improve the accessibility of cellulose for the gut microbiota: the linear SCFA concentration produced by fermentation of DC with DP 59 AGU and 32 AGU was 2.6 and 1.8 times higher compared to unmodified MC. Contrary to the breakdown by the CTec2 enzyme blend, the microbiota in this pooled inoculum could access the DC better than the AC. Furthermore, the slightly lower crystallinity of DC59 resulted in a slightly higher fermentation degree for DC59 than for DC32. The highest SCFA production, however, was obtained upon the addition of ADC to the fecal pool, with a linear SCFA concentration of 41.5 ± 6.4 mmol/L at the end of incubation. By reducing both the degree of polymerization and crystallinity of MC, the formation of linear SCFA by fermentation could be multiplied by a factor of 4.2. Based on the difference in mass of linear SCFA between the blank and ADC-enriched inoculum at 48 h, a minimal degree of fermentation (MDOF) of 45.8 ± 10.9% could be derived for the ADC25 sample, while this was only 7.6 ± 0.9% for MC. This MDOF is an underestimation of the actual fermentability since it only takes into account the mass of linear SCFA as a fermentation product. Furthermore, adding ADC to the fecal pool resulted in the largest pH drop, from 6.57 ± 0.01 to 5.67 ± 0.08 (Figure 4b). In vivo, such a pH drop could be associated with different physiological benefits, such as the repression of pathogen growth and proteolytic fermentation [9].

**Figure A2:**
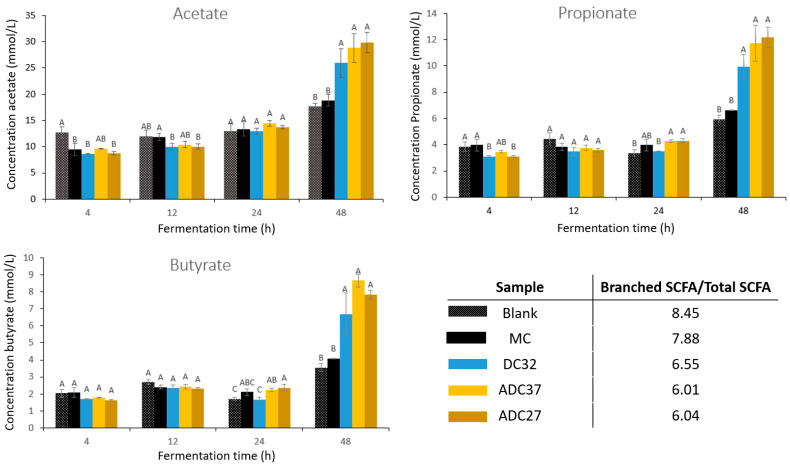
Acetate, butyrate and propionate concentrations, and the relative concentrations of branched SCFA (after 48 h) of cellulose-enriched fecal inocula in the second batch in vitro fermentation with pooled human feces, in function of incubation time. DCxx or ADCxx represents the fecal inoculum with the addition of a depolymerized cellulose or amorphized depolymerized cellulose with an average DP of xx. Different letters indicate significant differences between the samples at a certain timepoint (*p* < 0.05).

In a second in vitro fermentation experiment, two different chain lengths of ADC were investigated (Figure 4c,d). Additionally, the ball mill posttreatment time for the ADC was reduced to 1 h, instead of 6 h. Although a different pool of human feces was used, the same trends could be observed for the fermentability of these modified celluloses: unmodified MC was only fermented to a minimal extent, while decreasing the DP of the cellulose resulted in higher production of linear SCFA from the cellulose, up to a factor of 5.4 for DC32 after 48 h. The highest linear SCFA production was found for ADC samples ADC27 and ADC37 (8.2 and 8.4 times higher than for MC, respectively). The small difference in avDP, 37 versus 27, did not induce a significant difference in the fermentability of the ADC sample. The ADC samples were fermented to at least 42.6 ± 3.6%, while the MDOF of MC was only 5.7 ± 0.2%. The enhanced fermentation resulted in a larger pH drop of the ADC37-enriched inoculum (pH 5.58) than the MC-enriched inoculum (pH 6.02) (Figure 4d). Furthermore, it was demonstrated in this trial that this decreased pH resulted in a lowered production of branched SCFA as well (Figure A2). MC addition reduced the relative amount of branched SCFA from 8.5% to 7.9%, but the addition of ADC caused a further decrease to 6.0%. This is the first indication of a lowered protein fermentation in the inocula.

Detailed analysis of the acetate, butyrate and propionate concentrations demonstrate that the relative amounts of butyrate and propionate also increased after 48 h upon the addition of ADC. The relative amount of butyrate in total linear SCFA was 13.1% for the blank fecal slurry, while this was 17.6% for the fecal slurry with ADC37 addition (Figure A2). This enhanced butyrate production suggests an additional physiological benefit since enhanced butyrate production is linked to a lower risk of colon inflammation and cancer [41].

In this second trial, DC and ADC samples were mainly fermented between 24 and 48 h, while the main cellulose fermentation happened between 8 and 24 h in the first trial. The presence or absence of easily accessible fibers in the starting inoculum of the trials partly explains this difference. This was hypothesized since a fast production of linear SCFA after 4 h was observed in the second trial, while it was absent in the first trial. Therefore, the microbial community in the inoculum needed more time in this trial to switch to cellulolytic fermentation metabolism than in the absence of other (more easily fermentable) carbohydrate fibers in the first trial.

**Figure 5:**
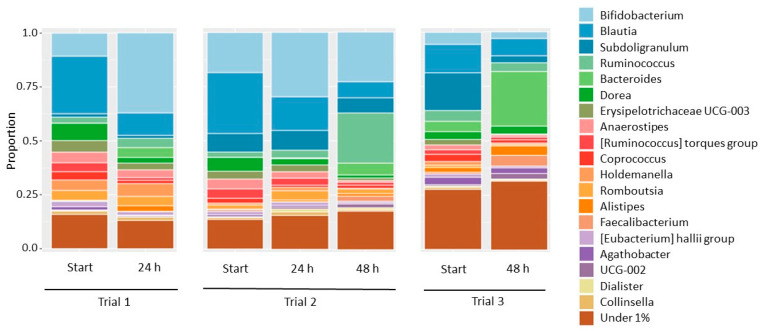
Microbial composition of starting, intermediate and end inocula of the different in vitro fermentation trials. The fecal inocula were enriched with ADC25 (Trial 1) and ADC27 (Trial 2, Trial 3).

A third trial (Figure 4e,f) showed a different fermentability behaviour for the ADC. No significant differences compared to the fermentability of unmodified MC were observed, and the pH decrease during the experiment was very limited for both the MC- and ADC-enriched inocula. Next to this trial, two other repetitions showed similar behaviour with no cellulose fermentation occurring for MC or ADC (data not shown). The starting microbial composition of the inoculum within every trial is different, of course, since other donors were used for the experiments. In Figure 5, the composition of the microbiome of the three in vitro trials is given at the genus level at the starting point of the experiment and after incubation with ADC.

For in vitro fermentation trials 1 and 2, the microbiome composition was dominated by *Bifidobacterium* and *Blautia* species. The proportion of *Bifidobacterium* species was lower for the first trial than for the second. Surprisingly, these *Bifidobacteria* seemed to dominate the cellulose fermentation in this first trial. After 24 h, when the ADC fermentation had already taken place, the DNA proportion from *Bifidobacterium* species increased from 10.7% to 37.0%, while other genera seemed to be suppressed. The fermentation of ADC might be driven by *Bifidobacteria*, but this enrichment can also be the result of the presence of glucose, which is released from cellulose by others. In the second trial, a different evolution of the microbial community was observed. While the microbial community was also enriched in *Bifidobacteria* at 24 h (when the ADC was not fermented yet), *Ruminococcus* species took the upper hand between 24 h and 48 h, which is the period that the ADC fermentation took place.

The microbiota in the third trial did not switch to a cellulolytic metabolism within the given time frame of 48 h. The microbial composition of the starting pool of this experiment was clearly less dominated by the *Bifidobacterium* and *Blautia* species than the ones from Trial 1 and Trial 2. Furthermore, during the experiment, *Bacteroides* dominated the medium instead of *Bifidobacterium* or *Ruminococcus*. Consequently, we can hypothesize that the switch to cellulose fermentation occurs only if specific specialized microorganisms are present, and the composition in the pool allows them to take the upper hand. Based on Trial 1 and 2, the authors hypothesize that this switch depends on the presence and abundance of specific *Ruminococcus* or *Bifidobacterium* species, but further research is needed to confirm this statement. Despite the comparable starting microbial community of those two trials, the fermentation of both ADC samples caused an enrichment of different microorganisms, demonstrating the complexity of this fermentation process.

## 4. Conclusions

The combination of ball milling with acid hydrolysis was demonstrated to be a valuable strategy for increasing the enzymatic accessibility of microcrystalline cellulose since it can selectively decrease the avDP and crystallinity of cellulose simultaneously. These modifications effectively resulted in an enhanced digestibility by a commercially available cellulase blend. Within an avDP range of 20–110 AGU, the avDP impacted the hydrolysability by this enzyme blend, once a low crystallinity was ensured. Furthermore, the enhanced accessibility of such amorphized depolymerized cellulose resulted in a higher fermentation degree compared to unmodified cellulose upon incubation with a pooled fecal inoculum from human subjects. With this modification, the minimal degree of fermentability of cellulose (based on the mass of SCFA produced from cellulose) within 48 h could be enhanced from 5% to 45%. This could be observed in two independent studies. However, other efforts did not show this enhanced cellulose fermentation. Microbial analyses of the fecal inocula revealed the complexity of cellulose fermentation in batch systems. Performing a detailed analysis of the cellulose fermentation metabolism in the human colon is, therefore, key to fully revealing the effect of DP and crystallinity of cellulose on fermentation in batch conditions. Until this is investigated, the authors would like to stress that the interpretation of in vitro fermentation results always has to be performed with caution, and total characterization of the microbial pool is always encouraged. However, we can conclude that engineering the properties of cellulose to high accessibility can improve the fermentation in the colon as well, be it under specific circumstances. With this work, the first step is taken towards a highly functional cellulose-type dietary fiber additive.

## 5. Patents

The use of amorphized depolymerized cellulose as partially fermentable dietary fiber is patented in EP2022/0784403 (not published).

## Data Availability

Data is available upon request via christophe.courtin@kuleuven.be.

## Appendix A

**Table A1:** Ball mill and hydrolysis conditions for different AC, DC and ADC samples, and their corresponding average degree of polymerization (avDP) and crystallinity index. ACxx, DCxx or ADCxx represents amorphized cellulose, depolymerized cellulose or amorphized depolymerized cellulose with an average degree of polymerization of xx.

	Ball Mill Time (min)	Ball Mill Speed (rpm)	Hydrolysis Time (h)	Hydrolysis Temperature (°C)	Ball Mill Time after Hydrolysis (min)	avDP (AGU)	Crystallinity Index
MC	-	-	-	-	-	168 ± 1	0.72
AC124	260	400	-	-	-	124 ± 1	<0.30
AC97	360	500	-	-	-	97 ± 0	<0.30
DC	360	500	16	120	-	32 ± 0	0.69
DC28	260	400	16	130	-	28 ± 1	0.62
DC42	260	400	16	110	-	42 ± 1	0.56
DC85	260	400	2	110	-	85 ± 1	0.47
DC104	260	400	2	90	-	104 ± 2	0.51
DC32	360	500	16	120	-	32 ± 0	0.69
DC59	360	500	3	100	-	59 ± 1	0.56
ADC25	360	500	16	120	360	25 ± 1	<0.30
ADC27	360	500	16	120	60	27 ± 1	<0.30
ADC37	60	500	16	120	60	37 ± 2	<0.30
